# Smell and Taste Function and Their Disturbances in Sjögren’s Syndrome

**DOI:** 10.3390/ijerph191912472

**Published:** 2022-09-30

**Authors:** Katarzyna Błochowiak

**Affiliations:** Department of Oral Surgery and Periodontology, Poznan University of Medical Sciences, 61-812 Poznan, Poland; kblochowiak@ump.edu.pl; Tel.: +48-608836850

**Keywords:** Sjögren’s syndrome, chemosensory dysfunction, taste, smell, burning mouth syndrome, xerostomia, autoimmune diseases, saliva, xerostomia

## Abstract

Chemosensory disorders are a possible disturbance in Sjögren’s syndrome (SS). The aim of the study is to comprehensively present chemosensory disorders in SS and to indicate their possible causes. The possible causes of taste and smell disorders in SS are changes in the structure of exocrine glands and their dysfunction, damage to receptors and weakening of their ability to regenerate, and neurological changes in the form of peripheral neuropathy and impaired cognitive function. Other postulated causes of chemosensory disorders are autoimmune mechanisms, adverse effects of drugs used in SS, and primary potentially SS-triggering viral infections. They are multifactorial and may occur independently of each other. The time of their onset and correlation with other disease symptoms may facilitate the determination of their primary cause in each patient. Awareness of chemosensory disorders in SS may help to ease their progress and eliminate other factors responsible for their more severe manifestation. In the prevention and treatment of chemosensory disorders in SS, the most important thing is to alleviate xerostomia and dryness in the nasal cavity and their effects in the form of chronic local inflammations, counteract receptor atrophy, and an implementation of appropriate neurological diagnosis and treatment.

## 1. Introduction

Sjogren’s syndrome (SS) is a chronic inflammatory autoimmune disease of unknown origin, involving primarily the exocrine glands, mainly salivary and lacrimal glands. It is characterized by periductal lymphocytic infiltrates of the exocrine glands leading to secretory dysfunction of the salivary and lacrimal glands in the form of xerostomia and xerophthalmia, respectively [1,2,3]. Decreased secretion of saliva and tears assessing by unstimulated salivary flow rate and Schirmer’s test, as well as the presence of lymphocytic infiltrates in the labial salivary glands (LSG) and its typical microscopic pattern, termed focal lymphocytic sialadenitis (FLS), are the main diagnostic criteria of SS [4,5]. Although SS can also trigger systemic, extraglandular changes, including pulmonary, renal, cutaneous, musculoskeletal, hematological, and neurological manifestations., the typical feature of SS is a local predominance of the pathological changes in the exocrine glands, such as the salivary glands [4].

Apart from the progressive exocrine gland damage and broadly distributed sicca symptoms, as well as extraglandular manifestations, some chemosensory dysfunctions in SS have been described so far [1,5,6,7].

Chemosensory function has serious implications for the preservation of oral and systemic health and an impact on quality of life. Limitations in the chemosensory function in SS may contribute to changing eating habits in the form of avoiding the consumption of certain foods and increased use of sugar and salt to compensate for the diminished sense of taste [8]. These changes in eating habits resulting from chemosensory disorders may increase the risk of civilization diseases, mainly cardiovascular disease, as well as predispose or worsen some oral cavity manifestations, such as caries or candidiasis that are the main SS complication in the oral cavity [9]. Moreover, some acidic foods may increase the pain and sensitivity of the oral mucosa resulting from decreased salivary flow. Previous studies revealed that chemosensory and secretory disorders in the form of hyposmia and xerostomia in SS patients caused changes in eating habits, including increased consumption of drinks and fats [10]. The most significant differences in the consumption of fat, fruits and vegetables were detected between hyposmic and normosmic SS patients. The intake of fruits, berries, and vegetables was lower in hyposmic SS patients than in normosmic SS patients [10]. Moreover, the same study revealed a lower intake of energy and total fat among the hyposmic SS patients. Another possible result of chemosensory disorders in SS is a lower intake of supplemental vitamin D in ageusic SS patients than in the normogeusic SS patients [10]. These changes in dietary composition induced by chemosensory disorders in SS may cause a limited local effect on the oral health as well as a more severe systemic impact on general health. In addition to significantly reducing the quality of life, chemosensory dysfunction may contribute to or worsen such adverse clinical conditions as depression, anorexia, and weight loss [11]. Therefore, awareness of these changes and their nature in SS may be helpful in the oral and systemic health improvement in SS patients and increasing their quality of life [1,8,11].

It is likely that SS-related chemosensory dysfunction has multifactorial origins and could be associated with reduced salivary flow, damage to peripheral sensory organs or damage to peripheral nerves and central nervous system [8,11]. Chemosensory dysfunction reported in SS is usually attributed to changes in salivary gland tissue morphology and xerostomia, but there is no direct relationship between the depth and nature of chemosensory dysfunction and the occurrence and severity of xerostomia.

The studies that were included in the presented review were identified using PubMed, Web of Science, and Google Scholar. The databases were searched using specific keywords, such as: “chemosensory dysfunction”, “Sjögren’s syndrome”, “autoimmune diseases”, “xerostomia”, “burning mouth syndrome”, “saliva”, “smell”, “taste”, “quality of life” in various combinations. We included literature written in English and published between 2000 and 2021.

Despite the significant progress in the research for chemosensory dysfunctions in SS, this review aims to explore and describe the most common chemosensory dysfunctions that have been reported in SS, as well as to introduce the possible sources of these disturbances. We draw attention to the nature, severity, and the potential triggering mechanisms.

## 2. Types of Chemosensory Dysfunctions in SS and Their Severity

Chemosensory disorders, which include olfactory and gustatory dysfunction, could manifest as reduced ability, distortion, or absence of the senses of taste and smell. Smell impairment can be classified as a hyposmia or diminished sense of smell, as a parosmia or aberrant odor perception and as a total loss of smell called an anosmia. In turn, taste dysfunction can be classified as a hypogeusia or diminished taste, a dysgeusia identified as distorted taste, as altered taste, known as an aliageusia, and ageusia or total loss of taste [7,11].

Among the chemosensory disorders, it is the taste and smell disorders that are most often reported in SS patients. These disturbances in SS patients were confirmed by both objective and subjective tests. In the SS group, the self-reported taste and smell scores on VAS were significantly lower than those of healthy controls [1]. Ageusia, hypogeusia, anosmia, and hyposmia were more frequent in the SS group than in healthy people [1,12]. Although the main types of taste and smell disorders were hyposmia and hypogeusia, anosmia, and ageusia were relatively common in the SS group [1]. It is postulated that anosmia is exclusively presented in the SS group compared to the control [12]. Furthermore, a high proportion of SS patients complained of dysgeusia [1]. Olfactory and gustatory functions significantly deteriorated in SS patients compared to age- and gender-matched controls, with approximately 50% of subjects suffering from hyposmia and 70% suffering from hypogeusia [5]. Similar results were obtained by Rusthen et al., who detected lower gustatory and olfactory scores in SS patients divided into two age subgroups—between 30–50 years old and 51–80 years old—than the controls divided into the same age subgroups [7]. Moreover, there is a positive correlation between smell and taste [5,7]. The opposite results were obtained by Gobeljić et al., who showed that the taste and smell disorders occur independently [1]. Moreover, they found that gustatory dysfunction is more frequent in SS patients than olfactory dysfunction [1]. These differences in results may be due to different test methods. Olfactory dysfunction in the SS group may increase with time, but a similar correlation is not observed for gustatory function. These differences in smell and taste perception with age could indicate various causes of these chemosensory dysfunctions [5]. The olfactory function is more sensitive to cognitive impairment that increases with age. Gustatory function is more associated with peripheral sensory impairment [7]. In their study, Kamel et al. reported that the smell threshold score was reduced by 1 point and the taste threshold score was reduced by 3.5 points in 28 patients with SS compared to 37 healthy participants. The most common chemosensory dysfunctions reported in this study were hyposmia and hypogeusia. Although the authors showed disturbances in the perception of both senses, by correlating their threshold with the physical and mental components of the Short Form 12 (SF-12) scale assessing the health-related quality of life, they observed that the taste threshold was correlated with physical and mental components of SF-12, while smell threshold positively correlated only with physical component [6]. These observations revealed the different nature of taste and smell dysfunction in SS. A similar taste dysfunction was observed by Al-Ezzi et al., who detected an evident taste disorder in 54% of SS patients and only in 8.3% of healthy volunteers [13]. Most chemosensory disorders are quantitative in the form of hyposmia or hypogeusia. Anosmia and ageusia are less common. Qualitative disturbances rarely occur alone. As a rule, they are correlated with other oral complaints [7]. In addition to general taste perception, some studies revealed various degrees of dysfunction for the main types of taste in SS patients. Sweet taste was the least affected, while bitter was the most impaired in the SS group [12]. Similar results were obtained by Kamel et al., who reported that within the SS group, the threshold for sweet taste was much less affected than the threshold for sour, salty and bitter [6].

Chemosensory dysfunction can be combined with burning sensations or numbness in the mouth. These sensory sensations can coexist with gustatory disturbances and they are described as burning mouth syndrome (BMS). BMS is a burning sensation mainly concerning the anterior two-thirds of the tongue, but other portions of the oral mucosa may be affected, too [7,14]. The symptoms are recurrent and last more than 2 h per day for at least 3 months [13]. According to other studies, BMS is diagnosed if burning sensations last for at least 4–6 months, and they may or may not be associated with normal clinical and laboratory findings [7]. The diagnosis of BMS is settled by exclusion because of the lack of the clinical manifestations [15,16]. The causes of BMS are unknown and can be a basis of BMS classification. Psychogenesis is the most important etiologic factor, but xerostomia and oral candidiasis are approved, too. Other known etiological factors are problems with dentures, parafunctions, such as clenching and tongue thrusting, hematological disorders, vitamin B complex deficiency, the climacteric, and undiagnosed diabetes. BMS may be classified as primary BMS in which the organic local or systemic causes cannot be identified, and neuropathological pathways are involved, and secondary, where local, systemic or psychological causes are identified [7]. This condition mainly affects menopausal and peri-menopausal females with the maximal rate observed at the age of 70–79 [17,18]. The symptoms are less prominent in the morning, worsen throughout the day, and stop completely at night [19]. Patients with BMS have a decreased unstimulated and stimulated salivary flow rate but to a lesser extent than patients with SS [20]. There is a strong correlation between the SS and the presence of BMS. Moreover, some SS patients may present co-existence of dysgeusia, halitosis, and BMS, described as burning sensations in the tongue (BST). There are mutual correlations between these oral complaints. Moreover, they are usually treated as main components of oral health and oral quality of life. Previous studies revealed a strong correlation between impaired olfactory and gustatory functions, BST and poor Oral health-related quality of life (OHRQoL) in SS [1]. Chemosensory and oral disorders and BMS reduced the patients’ quality of life assessed by the short-form Oral Health Impact Profile (OHIP-14). Scores in all domains of OHIP-14 including functional limitation, physical limitation, psychological limitation and social limitation were higher in SS patients compared to controls [1]. It seems that oral dryness, xerostomia, BMS and chemosensory disorders coexist in SS and mutually decrease OHRQoL [1,5]. It is interesting that BST did not correlate with saliva secretion [7]. A total of 42% of SS patients who reported BST described this problem as accompanying food intake, especially spicy and sour food. In turn, accompanying dysgeusia was described as a metallic, sour, bitter, harsh or rotten taste [7]. BST did not change with age, the disease duration, or the medications taken [7]. There is a strong relationship between BMS and SS. Gobeljić et al. reported that nearly half of patients with SS presented with BST [1]. The majority of SS patients experienced a burning sensation on the tongue during meals and 39% of them reported a sour taste sensation as a type of BST [1]. These findings confirmed that both SS and BMS could pre sent the same cause and nature of chemosensory disorders and that should be mutually described. Table 1 comprehensively presents and summarizes the findings related to taste and smell disorders in SS.

Although SS induced chemosensory disorders are combined with oral disorders and they may decrease OHRQoL, however some previous studies noticed that chemosensory impairment may also impact on selected components of general quality of life, including mental and physical components, anxiety, and depression [21]. Limitations in daily activities and functioning in SS induced by chemosensory disorders may worsen general quality of life and the fighting of them should be a part of the comprehensive treatment of SS patients.

## 3. Causes of Chemosensory Dysfunction in SS

Multiple factors could potentially contribute to the impairment of chemosensory perception in SS patients compared to age- and gender-matched controls. This reflects the rich symptomatology of SS and its involvement in the function of chemoreceptors, peripheral nerves, cognitive functions, and central nervous system functioning, as well as qualitative and quantitative changes in the oral and nasal cavities secretions. It also appears that some clinical symptoms developing in the course of the disease in the immediate vicinity of the taste and smell chemoreceptors in the oral and nasal cavities may exacerbate the chemosensory dysfunction or damage the chemoreceptors themselves. Furthermore, the therapies used in SS can worsen the perception of taste and smell. The possible cause of chemosensory disorders is indicated by the time of their appearance in relation to other SS symptoms. Their delayed occurrence may be correlated with the progressive destruction of the exocrine glands, mainly salivary glands and secretory glands in the nasal cavity, and a reduction in their secretory function or the destruction of chemoreceptors. On the other hand, the early appearance of disturbances in the perception of taste or smell may be the result of the involvement of nervous structures in SS or of direct mechanisms causing the disease. In addition, the disturbances in the sense of smell and taste may occur independently of each other or may be of different intensity. The chemosensory disorders observed in SS also show different progression with age, which may be a significant factor modulating the severity of these disorders. Taking these clinical observations into account, it seems that there is no universal mechanism that leads to chemosensory disorders in SS. Moreover, disturbances in the perception of smell and taste may be of a different origin, even in the same patients with SS.

### 3.1. Exocrine Dysfunction Degree and Alternation in Secretion Composition

Traditionally, chemosensory disorders in SS are attributed to xerostomia and nasal dryness, especially as generalized dryness may be the only symptom of the disease, and worsens over time. Moreover, a correlation not only between the saliva and mucus secretion but also between the dryness of the eye and the perception of smell was reported in the SS group [22]. Topan et al. reported the relationship between dry eye tests and olfactory function parameters and showed that they were moderately correlated. Schirmer’s test, tear break-up time (TBUT) test and ocular surface staining (OSS) score showed significant correlations with the odor threshold, odor identification score, Connecticut Chemosensory Clinical Research Center (CCCRC) score and olfaction Visual Analog Scale (VAS) score [22]. Olfactory dysfunction was more remarkable in pSS patients with moderate-severe OSS [22]. These findings revealed the existence of mutual relationship between chemosensory disorders and generalized exocrinopathy in SS. Lymphocytic infiltrates in the nasal and salivary glands accompanying SS progressively destroy the secretory epithelium of the glands, impairing their secretory functions. It is estimated that if xerostomia only occurs in approximately 30% of SS patients in the early stages of the disease, after 10 years, nearly 80% of SS patients experience symptoms of dry mouth [23]. However, progressive gland damage is not always correlated with progressive impairment of chemosensory function, indicating the existence of other mechanisms responsible for the disturbance to smell and taste. Some patients presenting with significant lymphocytic infiltrates in the exocrine glands and severe dry mouth and dry nose do not report any deficits in the perception of taste or smell even with time. The association between the reduced exocrine glands secretion and dryness is especially postulated with regard to taste perception. The basis of the reduced taste acuity in SS patients is the reduced ability to transferring signals to the taste buds. Saliva is a natural carrier for flavors and transports them to the chemoreceptors. Taste stimulants require salivary secretion to get to the taste buds. Salivary carrier proteins assist in transporting taste stimulants to the receptors [11,24]. Thus, xerostomia significantly reduces the access of stimuli to receptors causing taste impairment in SS patients. The relationship between decreased salivation and taste disturbances is also evidenced by reduced taste perception in people with symptoms of dry mouth who do not meet the SS criteria, as it has been presented in the study conducted by Negoro et al. [25]. Xerostomia and decreased taste acuity were detected in both the SS group and non-SS patients complaining of dry mouth or dry eye. Furthermore, these findings were confirmed by an electrogustometric examination, as well as by the filter paper disc method, indicating no significantly differences between these groups [25]. There were no differences in serum zinc protein levels described as a main gustatory proteins in both groups. It is worth noting that while in the non-SS group there was no relationship between saliva flow and the state of the papillae of the tongue, in the SS group, reduced salivation correlated with the atrophy of the papilla of the tongue, which may have a direct impact on the state and number of taste receptors and taste disturbances in SS [24]. It seems that the atrophy of the papilla of the tongue is more due to lymphatic infiltration than to decreased salivation [26]. These morphological changes in papillae point to another source of decreased taste perception in SS accompanying dry mouth. While the reduction of saliva secretion limits the direct short-term contact of food substances dissolved in saliva to the taste buds, reducing the flavors perception, low salivary flow reduces the regeneration of receptor sites and the ability to defend against bacterial infection, thermal and mechanical stress. This mechanism is long-standing and more SS-specific than simple chemical contact between flavors dissolved in saliva and taste buds [24]. Xerostomia in SS does not provide taste receptor site regeneration. Moreover, chemical changes in saliva composition observed in SS explain alternations in taste perception described as dysgeusia. Not only the amount of saliva but also changes in its composition are responsible for changes in taste perception. Changes in the activity of selected salivary digestive enzymes and decreased levels of proline-rich proteins result in an altered perception of bitter and sweet tastes [24]. A close relationship between salivary flow and taste perception was confirmed in a study conducted by Singh et al., who found that a significantly greater proportion of both SS patients and non-SS patients with dry mouth had ageusia, dysgeusia, and BMS compared to healthy controls [21]. Metallic taste dysgeusia was the most common complaint in both SS and non-SS groups [21]. On the other hand, there were no significant differences in median gustatory scores between the non-SS and control groups [21]. The same authors found a significant correlation between unstimulated whole salivary flow and stimulated whole saliva flow values and dysgeusia, taste score, and BMS in the SS group, the non-SS group, and healthy controls [2]. The theory related to a correlation between salivation and chemosensory dysfunction was not confirmed by Al-Ezzi et al., who did not find any association between taste acuity and oral dryness [13]. Furthermore, oral dryness was not a good predictor of the neurosensory threshold of taste in the SS group. According to Gomez et al., pSS patients even with severely reduced salivary flow were able to recognize the four basic tastes at suprathreshold concentrations [27]. Even the lowest amount of saliva or the longest time with the disease did not reflect the highest detection or recognition thresholds for a particular taste. The detection thresholds for the sweet, sour, and bitter tastes were higher in pSS patients compared to healthy control but not in the same degree. These findings ruled out any effect of the reduced salivary flow on the taste acuity [27]. Similar results were obtained in the study conducted by Rusthen et al., who did not detect any correlations between salivary secretion and the presence of oral disorders including BMS, smell and taste impairments [7]. On the other hand, an argument that could confirm the dependence of taste disturbances on the degree of damage to the salivary glands and the severity of xerostomia is the gradual increase in disturbances in the perception of taste. In addition, changes in the composition of saliva and nasal secretion impair an important defensive barrier against infections. Reducing the physiological mechanisms of protection of the epithelium lining, and the oral and nasal cavities by means of a decrease in the level of mucus secretion, and changing its composition predisposes it to epithelial inflammation and its post-inflammatory remodeling. Electron microscopy of the nasal epithelium revealed very thin, atrophic, and breached basal lamina and growth of dense collagen bundles in the submucosa and involvement of the epithelial surface. Predominant dryness of the nose and recurrent rhinosinusitis, as well as more frequent epistaxis and crusting in the nasal cavity observed in SS can potentially cause permanent damage to the ability to perceive odors. Therefore, it seems important to investigate whether the structural changes in the epithelium in the olfactory part of the nasal cavity occurring in SS have sufficient potential to impair the sense of smell. Although some studies have revealed the occurrence of nasal septal perforation and increased dryness of the nose and hyposmia confirmed in the Smell Threshold Test, the dependence of smell disorders on changes in the mucosa of the nasal cavity seems less pronounced than in the case of taste sensation [28]. In a study conducted by Eren et al., the authors reported that although in SS patients the intranasal Schirmer test was decreased and nasal dryness was more pronounced compared to controls, there were no differences in olfactory functions between SS patients and controls. They only found a trend toward a positive correlation between olfactory function and the nasal Schirmer score but without statistical significance. Moreover, there was no relationship between mucociliary clearance and the Schirmer test score [29]. Similar results were obtained by Midilli et al., who reported that nasal findings in SS were insignificant and mild compared to healthy participants even in patients with severe oral or ocular findings. There were no severe smell disorders in the SS group. It is worth noticing that detected olfactory disorders were associated with nasal polyposis rather than mild mucosal involvement in SS patients [30]. It is estimated that 41% to 69% of olfactory impairment is correlated with the incidence of polyps in the nose [11]. These findings confirmed that nasal cavity humidity is not related to olfactory function.

### 3.2. Peripheral Sensory Impairment and Cranial Nerve Involvement

The other postulated cause of chemosensory disturbances in SS is involvement of the central or peripheral nervous system. The pathomechanism of nervous system impairment in SS includes epineural infiltration by inflammatory cells, vascular injury mediated by autoantibodies, and ischemia due to small vessel vasculitis [31]. One of the most common neurological manifestations in SS are neuropathies. The prevalence of neuropathy in SS ranges from 2–60% [32]. It presents in various types and diverse responses to the applied treatment indicating a complex pathomechanism responsible for its occurrence [32,33]. The involvement of the peripheral nervous system in SS may demonstrate as trigeminal neuropathy, multiple cranial neuropathy, or simple pain neuropathy [34]. Previous studies revealed that an increase in neuropathy severity may be accompanied by a decreased ability to identify odors [35]. It seems that among various types of peripheral neuropathies in SS, damage to the cranial nerves is the most important factor for the development of chemosensory disorders. It is estimated that damage to the cranial nerves occurs in 39% of SS patients compared to people without SS [36]. Some previous research noticed the close relationship between chemosensory disorders and the involvement of cranial nerves [32,37]. Most common cranial neuropathy in SS is trigeminal. It develops slowly with the progression of SS and it occurs rarely in the initial stage of the disease [34]. Moreover, the degree of trigeminal nerve involvement may correlate with chemosensory disfunction [33]. Similar correlations were found in BMS, indicating the existence of trigeminal small fiber neuropathy [35]. Another possible relationship between dysgeusia associated with BMS and peripheral neuropathies is related to damage to or hypofunction of the chorda tympani. The mechanism of cranial nerve involvement may be either by continuity from nasal and oral cavity or by vasculitis of the small blood vessels surrounding the cranial nerves, resulting in mononeuritis multiplex. This possible pathway has been described in Wegener’s granulomatosis but may be transmitted in SS [37]. Furthermore, perivascular inflammatory infiltrates could damage cranial nerves in SS. In turn, antibodies seem not to have any impact on development of neuropathies in SS. These observations were confirmed by Al-Ezzi et al., who demonstrated that taste impairment participated to some extent in SS-associated neuropathy [13]. The same authors reported that the taste function assessed on the three sites of the anterior 2/3 tongue with a electrogustometer was statistically significantly impaired in the SS group compared to the controls. The average score of the neurosensory threshold was three times higher in the SS group than in the healthy group [13]. These findings suggest a potential influence of lingual neuropathy in the occurrence of dysgeusia in SS patients. Interestingly, patients with an evident taste conduction disturbance did not report taste impairment in subjective tests. This indicates that taste dysfunction may be more common in SS than is indicated by subjective tests [13,36].

### 3.3. Central Nervous System Dysfunction and Cognitive Disorders

Apart from peripheral neuropathies, cranial nerve damage, and autonomic nervous system involvement, SS also induces changes in the central nervous system. They may exert an indirect influence on chemosensory disorders through a mechanism similar to that described for multiple sclerosis. Although central nervous system involvement is much less common in SS and ranges from 2–25%, we cannot exclude its indirect effect on olfactory and gustatory function [33]. SS may be characterized by a higher frequency of cognitive impairment, depression, anxiety, and diseases of the central nervous system [38]. Cognitive changes and depression can exacerbate chemosensory disturbances. Central nervous system involvement and the anxiety and depression observed in some autoimmune diseases, such as lupus erythematosus, increase a risk of olfactory disorders development. The presence of olfactory dysfunction is correlated with the radiological symptoms of central nervous system involvement, including smaller left hipocampus volume, and smaller left and right amygdalae volume [39]. Cognitive disorders are detected in 55% of SS patients and their severity ranges from severe in 17% to mild in 38% of SS patients [40]. This rate could be even higher in SS patients with symptoms of small fiber neuropathy or with chronic pain syndrome [40]. Cognitive disorders are most often correlated with the lower quality of life reported by patients and with disease activity. Although objective studies do not confirm a direct correlation between quality of life and taste disturbances, depression and cognitive disturbances intensify the sensations of taste disturbances in subjective tests [13]. Previous studies concerning taste and smell function in patients with cognitive impairment revealed that even mild cognitive disorders may be associated with decreased chemosensory function. Taste disorders in elderly people with cognitive decline are independent of factors affecting taste, such as salivation, zinc levels, or prescription drugs. This points to the potential influence of cognitive impairment in chemosensory function in SS. However, these chemosensory disorders occur only in the most advanced types of cognitive impairment, such as dementia, and they are not present in mild cognitive impairment [41]. Usually, SS does not induce such a severe type of cognitive impairment. In addition, cognitive disorders increase with age and therefore, a large group of young SS patients will not demonstrate them. Therefore, among the potential sources of chemosensory disorders in SS, cognitive disorders seem to be of limited importance. They may have the effect of reducing recognition of selected flavors rather than the overall taste threshold. It is postulated that cognitive impairment has the greatest impact on distinguishing umami taste, which results in decreased appetite in people with cognitive impairment [41]. Moreover, it is worth emphasizing that chemosensory disorders in people with cognitive impairment are detected in objective tests but are rarely reported by patients in subjective tests. The dependence of chemosensory dysfunctions on cognitive dysfunctions in SS is difficult to assess due to the possible contribution of other factors causing cognitive disorders that increase with age, but it is estimated that 5% of SS patients suffer from dementia directly related to SS. It is SS-specific autoimmune dementia that is not associated with changes in brain MRI [42].

### 3.4. Chemosensory Dysfunction as an Effect of Autoimmune Diseases

SS is defined as an autoimmune disease. Although the impact of immune disorders in SS as a potential cause of chemosensory disorders has not been studied so far, observations regarding other autoimmune diseases may be a common and universal way to explain these disorders. Olfactory and taste disturbances have been reported so far in autoimmune diseases, such as acute disseminated encephalomyelitis (ADE), allergic rhinitis, asthma, autoimmune pancreatitis, Churg–Strauss syndrome, Behcet’s disease, fibromyalgia, Wegener’s granulomatosis, inflammatory bowel disease, multiple sclerosis, psoriasis vulgaris, Mikulicz disease, lupus, scleroderma, and rheumatoid arthritis [43,44,45,46]. In many of these diseases, chemosensory disorders are not reported by patients or are very discreet and detected only by objective tests. Moreover, this relationship is more expressed in olfactory function than in gustatory function. It is postulated that olfactory disfunction correlates with disease activity in SS [12]. The first mechanism explaining the existence of chemosensory disorders in autoimmune diseases is the direct influence of the immune system on the respiratory and oral epithelium. Olfactory and oral epithelium constitute the first line of immune defense against xenobiotics. However, lymphocytic infiltrates, which are a natural response of the immune system, lead to impaired epithelial function, including its chemosensory function. Moreover, chronic inflammation of the epithelium may induce permanent structural changes in the olfactory bulb and in the composition of the papilla of the tongue as was described in allergic rhinitis, Behcet’s disease, and inflammatory bowel disease [43]. A similar mechanism of decreased olfactory function was detected in pemphigus vulgaris, where immuno-induced nasal lesions resulted in worse olfactory scores [47]. Moreover, it is postulated that the impaired sense of smell in patients with Mikulicz’s disease is a direct result of the infiltration of nasal mucosa by IgG4-positive cells [48]. However, previous studies revealed that autoimmune diseases, particularly with central or peripheral nervous system involvement, may be more predisposed to chemosensory disruption than epithelial inflammation. This is related to the direct influence of immune cells on the functions of the nervous system. Infiltration of activated immune cells leads to the axonal loss, demyelination of nerve fibers, and consequently, to chemosensory dysfunction. It seems that nervous system involvement and the immuno induced axonal loss could be a universal pathway of chemosensory dysfunction in rheumatic diseases, such as lupus, rheumatoid arthritis, scleroderma, and SS [43,49]. In addition to the immuno-induced epithelial lesions and the direct influence of immune cell in the neural function, resulting in olfactory and gustatory disfunction, another potential cause of chemosensory disorders in immune diseases are pro-inflammatory cytokines. One of the most potent cytokines is tumor necrosis factor (TNF) [50]. It impairs olfactory function and suppresses neuroepithelial regeneration. The more active the disease course and the higher the level of inflammatory parameters, the more severe the chemosensory dysfunction [50]. These observations have been supported by previous studies, which revealed that olfactory function assessed by the Connecticut Chemosensory Clinical Research Center method in patients with systemic lupus erythematosus (SLE) was decreased compared to healthy controls. Moreover, this disruption correlated with disease activity and anti-ribosomal protein antibody serum levels. With a more active disease course and higher serum levels of anti-ribosomal P protein antibody, the olfactory function in SLE patients is weaker. Furthermore, olfactory disorders are not always correlated with the central nervous system impairment, but they are an important determinant of disease activity. These findings indicate a strong relationship between immune disturbances and the chemosensory function [39,51]. The findings related to SLE patients were confirmed in the SS group. Xue et al. revealed that the disease activity in SS exhibited by the EULAR Sjögren’s Syndrome Disease Activity Index (ESSDAI) negatively correlated with olfactory function. Smell threshold scores were decreased in SS patients with anti-SSA antibody or anti-nuclear antibody compared with those without those autoantibodies. Moreover, olfactory identification and memory scores were decreased in pSS patients with concurrent thyroid dysfunction or hypocomplementemia, indicating a mutual relation between olfactory disfunction and autoimmune diseases [12]. Previous studies have postulated that in autoimmune diseases taste disfunction is associated with local damage in olfactory disfunction that is correlated with disease activity. Mouse models of autoimmune disease revealed that selected taste receptor cells could be affected, leading to taste disorders. This animal model resembling SLE and SS exhibited characteristics of inflammation of the taste epithelium, including infiltration of T lymphocytes and increased levels of some proinflammatory cytokines, such as IL-10, tumor necrosis factor -α (TNF-α), and interferon γ (INF-γ). Long-term chronic inflammation induced changes in taste bud structure and disrupted their integrity. Moreover, taste buds renewal is inhibited in a SS-specific animal model and the number of gustducin-positive taste receptor cells is reduced [52]. These findings confirmed in the SS animal model indicated that responses to bitter, sweet, and umami taste compounds are reduced in the greatest extent in SS. This weaker response to other tastes is the result of the interaction of other factors independent of autoimmunity [52].

### 3.5. Other Causes of Chemosensory Dysfunction

Drugs used in the treatment of SS might also be other potential causes of taste and smell dysfunctions. This relationship is postulated in elder persons in particular. The use of many medications in the elderly is directly related to the occurrence of BMS, dysgeusia and xerostomia. Drugs disrupt the physiological mechanisms that protect against the negative consequences of decreased salivation occurring with age [53]. Medicines can worsen the sense of taste and smell. However, they do not appear to have a significant effect in producing these disorders. Rushten at al. detected a weak correlation between olfactory/gustatory scores and the number of medicines [7]. Immunosuppressants are drugs that are known to affect the sense of taste and are also used in the treatment of SS. Although there are no studies on their effect on taste perception in SS, studies in other patient groups have shown that immunosuppressants reduce taste perception. Patients reported a metallic taste in their mouths and objective tests detected dysgesia or hypogesia. However, the effect of the drugs on the weakening of taste gradually decreased over time and after 6 months came closer to the values of the control group [54]. The most severe taste alternation were detected in the perception of umami and salty tastes [55]. It seems that immunosuppressive drugs that are used in SS therapy in even lower doses than in cancer chemotherapy may only temporarily weaken the sense of taste. In the SS group, there were no correlations between olfactory function and oral steroids, hydroxychloroquine and immunosuppresants [12]. Similar results were obtained in rheumatoid arthritis (RA) patients taking different drugs where there was no significant changes in olfactory and gustatory function. There was only a tendency in decreased smell and taste function in RA patients taking non-steroidal anti-inflammatory drugs, TNFα inhibitors, other biologics, leflunomide and methotrexate [56]. It seems that there is a modulating effect of medicines in the perception of taste and smell, especially in a self-reported olfactory and gustatory test. The most important modulating chemosensory function are anti-inflammatory and immunosuppressive drugs commonly used in the treatment of SS. The main medications responsible for impaired smell perception are diclofenac, dexamethasone, gold, hydrocortisone, and methotrexate [10]. Moreover, it is postulated that some oral low-dose corticosteroids decrease olfactory function [56]. Multiregression analysis revealed the negative effect of some painkillers commonly used in SS, such as naproxen, diclofenac, paracetamol, and aspirin on gustatory function [13]. These results indicate the possible influence of the different drugs used as one of the many taste and olfactory deterioration factors, especially when multiple drugs are used and other modulating factors are present. However, the effect of the cumulative dose on the impairment of the sense of smell and taste and their long-term use cannot be taken into account, as there is no correlation between the damage to the senses of smell and taste with the duration of the disease and the duration of therapy [7].

It cannot be ruled out that just as the primary cause of SS is a viral infection in genetically predisposed patients, the olfactory disorders observed in these patients are a consequence of the primary viral infection destroying peripheral olfactory receptors and neural pathways to the brain [11], especially since the correlation of olfactory disorders with previous viral infection is high and estimated at 14% to 25% of all cases [11]. Even the most common viruses that are responsible for colds may cause postviral olfactory dysfunction. They include coronaviruses, influenza viruses, parainfluenza viruses, respiratory syncytial viruses, adenoviruses, and enteroviruses, and they can account for at least 70% or more of viral upper respiratory infections [57]. Moreover, these viruses were detected in the nasal discharge of patients with olfactory dysfunction after primary upper respiratory infections. It is postulated that some of the aforementioned viruses have the potential to impair olfaction not only through nasal obstruction but also through other mechanisms [57]. These mechanisms are responsible for persistent olfactory disorders after cold symptoms have resolved. One suggested pathomechanism of persistent olfactory disorder induced by viral infection is impairment of neuronal apoptosis, resulting in impaired proliferation of the olfactory epithelium, and causing its impaired regeneration [58]. Although these viruses are not directly involved in SS, they should be treated as potential trigger factors of both SS and accompanying olfactory disorders. Moreover, some studies demonstrated a significant increased risk of SS developing in individuals with a history of multiple infections independently of the type of pathogens [59]. In particular, infections of the respiratory tract and its common viruses are associated with an approximately three-fold risk of developing SS and could be indirectly associated with olfactory disorders [59]. Previous studies suggested that Epstein–Barr virus, human T lymphotropic virus type I, hepatitis B and C viruses, cytomegalovirus, and the mumps virus are potential trigger viruses in SS [59]. These viruses lead to hyperstimulation of the immune system and autoimmunity, reflected in the increased level of SS-specific antibodies, which indicates that the source of olfactory disorders in SS is more of the autoimmunity process than direct damage to the respiratory receptors, which often occurs as a sequencing of upper respiratory tract infections. Changes in circulating leukocyte subsets and an extensive increase in the concentration of pro-inflammatory cytokines in SS are similar to those seen in SARS-CoV-2 infection [60]. A similar pathomechanism of chemosensory disorders in both SS and SARS-CoV-2 is evidenced by the fact that the olfactory disorders in SARS-CoV-2 are often isolated and without any prior connection with an upper respiratory tract infection. In this pathomechanism, attention is paid to the possible simultaneous influence of pro-inflammatory cytokines on the immune system and the nervous system, direct destruction of nerve cells by pro-inflammatory cytokines [60]. The effect of viral infection on impairment of taste is less well understood than impairment of smell. There is no direct relationship between dysgeusia and viral infection, including infections associated with the pathogenesis of SS. However, some viral infections, including hepatitis E, cytomegalovirus, are involved in possible taste disorder development through disrupting the normal cell turnover of taste buds [61]. It seems that the influence of a viral infection on taste perception is indirect and occurs not through autoimmunity, as in the case of the sense of smell but through damage to taste receptors by viral infection.

The other potential cause of taste and smell dysfunction is inflammation in the oral and nasal cavity. Lack of antibacterial salivary proteins accompanying SS can predispose to many opportunistic infections and microinjuries. There is a strong correlation between the occurrence of hypogeusia or dysgeusia and mouth infections. Detection of Toll-like receptors (TLRs) and interferon (IFN) signaling pathways in the taste buds and their stimulation by viral and bacterial infections in the oral cavity pointed to other potential sources of taste disorders through changes in gene expression in the taste cells [62]. Local infection induces disorders in physiological cell turnover in the taste buds and alters the representation of selected taste cells [63]. In addition to denervation of the peripheral gustatory nerves, inflammation appears to be the most important local factor leading to taste disturbances. A special role in this pathomechanism is attributed to selected pro-inflammatory cytokines, such as tumor necrosis factor (TNF)-α and interleukin (IL)-6, which are also involved in neurogenesis, regulation of progenitor cell proliferation, and neural differentiation. They are produced by some taste bud cells in inflammatory conditions after lipopolysaccharide (LPR) stimulation. LPS-induced inflammation inhibits taste progenitor cell proliferation and results in a reduced number of newborn cells entering the taste buds. Moreover, inflammation shortens the average turnover period of taste bud cells, leading to possible taste disorders [64]. It is worth noting that inflammation modulates our sensitivity to specific tastes, while it only slightly affects the threshold of perceiving the taste. This was partially confirmed in study conducted by Feng et al., who suggested that TNF plays a role in sensitizing bitter taste responses. Elevated levels of TNF during inflammation contributed to a persistent bitter taste sensation in some patients with taste abnormalities [65]. Another possible source of taste disorders in SS is mucositis. Mucositis induced by xerostomia can reduce the perception of bitter and salty taste. A similar mechanism is observed after radiotherapy, and a possible explanation for its causes is a blockage of the taste buds by neighboring epithelial cells [66]. The role of inflammation and pro-inflammatory cytokines in decreased olfactory function have been widely discussed in previous studies. Close correlations were present in patients with rhinosinusitis and acute rhinitis [67,68]. However, these olfactory disorders are usually temporary and they are limited to acute stages of inflammation. Short courses of inflammation do not induce severe changes in olfactory epithelium. Moreover, while xerostomia is an important factor contributing to infection and subsequent dysgeusia in SS, there are no convincing data that dryness of the nose in SS is associated with a greater frequency of inflammation of the nasal mucosa and olfactory disorders. It seems that nasal inflammation has a limited impact on olfaction. However, the incidence of chronic rhinosinusitis is significantly higher in patients with SS than in healthy controls [69]. Chronic and allergic rhinosinusitis could induce constant changes in nasal epithelium and polyps development, which are important factors contributing to olfactory disorders.

## 4. Conclusions

Chemosensory disorders in SS may be multifactorial and may occur independently of each other. The time of their onset and correlation with other disease symptoms may facilitate the determination of their primary cause in each patient. This, in turn, may help to ease their progress and eliminate other factors responsible for the more severe manifestation of chemosensory disorders. In general guidelines for SS patients affected with chemosensory disorders, the most important issue is the awareness of taste and smell disorder occurrence in SS and their possible correlations with peripheral nervous system involvement and oral and nasal dryness, early diagnosing of taste and smell dysfunctions and fighting infections in the nasal and oral cavity. To answer these needs, novel and easily available methods were implemented in the diagnosing and monitoring of chemosensory disorders that could be adapted to SS individuals. They can include chemosensory function assessment with SNOT-22 that is filled out by the patients digitally via a QR-code as providing by the software ENT-Statistics and more objective methods, such as chemosensory event-related potentials and extended Sniffin’ Sticks test battery based on odor-containing felt tips [70,71,72]. In the prevention and treatment of chemosensory disorders in SS, the most important thing is to alleviate xerostomia and dryness in the nasal cavity and their effects in the form of chronic local inflammations, counteract receptor atrophy and the implementation of appropriate neurological diagnosis and treatment.

## Figures and Tables

**Table 1:** The findings related to taste and smell disorders in SS.

Chemosensory Dysfunction	Cohort (*n*)	Methods	Results	References
**Olfactory function**	SS:58 HS:55	Sniffin Sticks tests VAS	Hyposmia (SS vs. HS 36.5% vs. 13.2%) Anosmia (SS vs HS 3.8% vs. 0%) Lower olfactory score in SS vs. HS (8.6 vs. 9.6)	Gobejić et al. [1]
	SS:28 HS:37	Smell Threshold Tests	The smell threshold was reduced by 1 point in SS compared with HS Hyposmia (SS vs. HS 43% vs. 19%)	Kamel et al. [6]
	SS:31 HS:33	Sniffin Sticks tests VAS	Hyposmia (SS vs. HS 29% vs. 9%) Anosmia (SS vs. HS 13% vs. 0%) Lower olfactory score in SS vs. HS (8.8 in SS vs. 10.7 in HS)	Rusthen et al. [7]
**Gustatory function**	SS:58 HS:55	Taste strips VAS	Ageusia for four basic tastes in SS: sweetness (SS vs HS 34.0% vs. 7.5%), sourness (SS vs. HS 10.6% vs. 0.0%), saltiness (SS vs. HS 10.0% vs. 5.7%), bitterness (SS vs. HS 19.1% vs. 1.9%) Dysgeusia (SS vs. HS 52.6% vs. 9.4%)	Gobejić et al. [1]
	SS:65 (female) HS:62	Taste strips VAS Electrogustomer	Taste dysfunction (SS vs. HS 54% vs. 8.3%) There was no correlation between taste function and oral dryness There was correlation between taste acuity and the neurosensory threshold	Al-Ezzi et al. [5]
	SS:28 HS:37	Taste strips	Taste threshold score was reduced by 3.5 points in SS compared with HS Hypogeusia (SS vs. HS 71% vs. 35%)	Kamel et al. [6]
	SS:31 HS:33	Taste test VAS	Lower gustatory score in SS vs. HS (18.9 in SS vs. 25.4 in HS) Hypogeusia (SS vs. HS 32% vs. 12%) Ageusia (SS vs. HS 19% vs. 0%)	Rusthen et al. [7]
	SS:58 Non-SS sicca patients 22 HS:57	Taste strips Questionnaires	Hypogeusia (25.9% SS vs. 9.1% non-SS sicca vs. 12.3% HS)	Singh et al. [21]
